# Progress of plant polyphenol extracts in treating depression by anti-neuroinflammatory mechanism: A review

**DOI:** 10.1097/MD.0000000000037151

**Published:** 2023-02-02

**Authors:** Yuting Guo, Yan Yang

**Affiliations:** a School of Health Preservation and Rehabilitation, Chengdu University of Traditional Chinese Medicine, Chengdu, China; b Medical Department, The Third Affiliated Hospital of Chengdu University of Traditional Chinese Medicine, Chengdu, China.

**Keywords:** depression, dietary polyphenols, neuroinflammation, prevention and control mechanisms, review

## Abstract

There is a growing body of evidence supporting the involvement of central nervous system inflammation in the pathophysiology of depression. Polyphenols are a diverse group of compounds known for their antioxidative and anti-inflammatory properties. They offer a promising and effective supplementary approach to alleviating neuropsychiatric symptoms associated with inflammation-induced depression. This paper provides a summary of the potential anti-neuroinflammatory mechanisms of plant polyphenol extracts against depression. This includes direct interference with inflammatory regulators and inhibition of the expression of pro-inflammatory cytokines. Additionally, it covers downregulating the expression of pro-inflammatory cytokines by altering protein kinases or affecting the activity of the signaling pathways that they activate. These pathways interfere with the conduction of signaling molecules, resulting in the destruction and reduced synthesis of all inflammatory mediators and cytokines. This reduces the apoptosis of neurons and plays a neuroprotective role. This paper provides a theoretical basis for the clinical application of plant polyphenols.

## 1. Introduction

Depression is a psychological disorder that is characterized by a pervasive low mood, anhedonia, and alterations in cognitive function. In severe cases, individuals may experience suicidal tendencies. In China, the lifetime prevalence of depression stands at 6.8%,^[1]^ and it is anticipated to emerge as the primary driver of disease burden by 2030. Depression is intricately linked with a constellation of factors, including monoamine activity, neurogenesis, neuronal function, synaptic plasticity, dysregulation of hormonal signaling within the hypothalamic-pituitary-adrenal axis, oxidative stress, and inflammatory processes.^[2]^

Multiple studies have shown that the immune system can regulate neurotransmission and neural circuit systems, thereby altering central nervous system (CNS) homeostasis, neurogenesis, and emotional control.^[3,4]^ In the current clinical landscape, most prescribed antidepressants are synthesized pharmaceuticals with limitations such as a restricted antidepressant spectrum, conspicuous adverse reactions, and a penchant for disease recurrence. By contrast, polyphenols originating from plants fall under the category of naturally occurring compounds and are further classified into phenolic acids, flavonoids, stilbenes, and lignans. Prior investigations have established their antidepressant efficacy through mechanisms that involve inhibiting neuronal apoptosis, stimulating adult neurogenesis, and reducing oxidative stress and inflammation.^[5]^ Consequently, there is a discernible trend towards the use of natural antidepressants derived from plants that meet the criteria of natural origin, heightened efficacy, and minimal toxicity. This article provides a comprehensive review of the mechanisms by which plant polyphenols affect central nervous inflammation in the context of depression, offering new avenues and concepts for the prevention and treatment of depressive disorders.

## 2. Depression and central nervous inflammation

**Figure 1.**
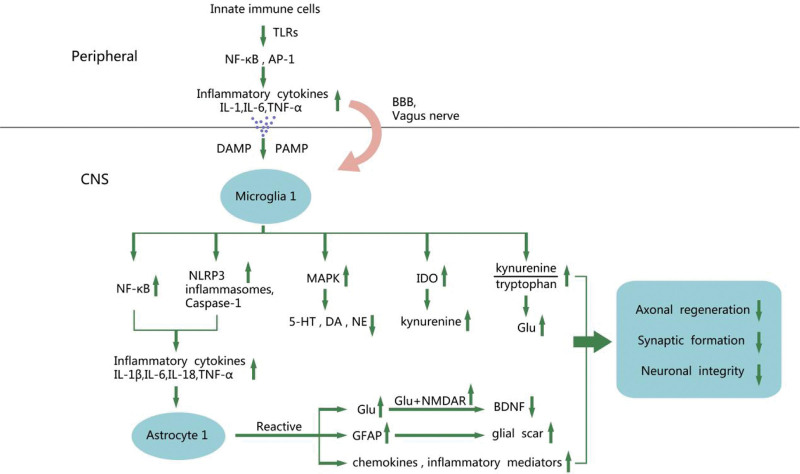
A schematic summary of mechanisms by which neuroinflammatory mechanisms of depression.

These receptors detect pathogen-associated molecular patterns, subsequently initiating inflammatory signaling pathways and synthesizing pro-inflammatory cytokines such as IL-1, IL-6, IL-18, and tumor necrosis factor (TNF-α).^[6]^ These pro-inflammatory cytokines induce changes in the endothelial cells comprising the blood-brain barrier (BBB), increasing permeability. The breakdown of the blood-brain barrier increases its permeability, allowing cytokines and immune cells to enter the brain. Within the brain, these entities activate microglia and astrocytes, ultimately producing inflammatory cytokines in a reverse manner.^[7]^ Together they lead to the development of neuroinflammation, which causes neuronal apoptosis (Fig. 1).

### 2.1. Microglia

Microglia represent the innate immune cells within CNS and are ubiquitously distributed throughout the brain and spinal cord. They function as an active immunological barrier in the CNS, serving as the foremost line of defense against neuroinflammatory processes within the CNS.^[8]^ Positron emission tomography examinations have revealed heightened microglial presence in the anterior cingulate cortex during episodes experienced by individuals with significant depression. This augmentation is accompanied by elevated indicators of microglial activation, as evidenced by increased translocator protein levels.^[9,10]^

Microglia initiate intracellular signaling pathways upon recognizing damage-associated molecular patterns or pathogen-associated molecular patterns on their cell membranes. These signaling pathways comprise nuclear factor kappa B (NF-κB) and NLRP3 (NOD-like receptor family, pyrin domain Containing) inflammasome, which can elicit immune regulation.^[11]^ They activate both classically activated (M1) and alternatively activated (M2) phenotypes. M1 microglia result in an increase in pro-inflammatory cytokines and the release of excessive nitric oxide, oxygen free radicals, and other inflammatory mediators, which directly lead to neurotoxic effects.^[12]^ In contrast, M2 microglia tend to express anti-inflammatory cytokines, arginase-1, and transforming growth factor-β1 (TGF-β1), thereby promoting nerve repair and the secretion of neurotrophic factors that support regeneration, ultimately reducing or inhibiting the inflammatory response.^[13]^ Microglia also regulate the metabolism of monoamines by activating mitogen-activated protein kinase (MAPK). This, in turn, increases the number and activity of presynaptic reuptake pumps, which reduces monoamines such as serotonin (5-HT), dopamine (DA), and norepinephrine (NE) in neuronal synapses. Microglia release also activates indoleamine 2,3 dioxygenase (IDO), which metabolizes tryptophan to kynurenine, ultimately leading to increased glutamate (Glu) release.^[6]^

The NLRP3 inflammasome constitutes a multimolecular complex comprising cytosolic NLRP3, the adaptor protein ASC, and pro-caspase-1. This complex is primarily expressed in central microglia and peripheral macrophages.^[14]^ Upon activation of NLRP3 by repetitive stress, it governs the activation of caspase-1, consequently facilitating the maturation of IL-1β and IL-18 within microglia.^[15]^ Furthermore, ATP binding to the purinergic P2X7 receptor leads to potassium efflux and the subsequent activation of the NLRP3 inflammasome.^[16]^ Notably, the gene encoding the P2X7 receptor is associated with the risk of depression.^[17]^ Neuroinflammation, which ensues from the activation of the P2X7-NLRP3 inflammasome cascade within microglia, represents a pivotal biological process in the pathophysiology of depression.

### 2.2. Astrocyte

In addition to microglia, astrocytes (AS) have emerged as pivotal contributors to the innate immune response within CNS against infections, neurodegenerative conditions, and injuries. Microglia exhibit a heightened sensitivity to pathogens or injuries and serve as the initial “pioneers” of activation. They subsequently trigger the reactivity of astrocytes (A1) by transmitting danger signals through shared molecular pathways.^[18]^ Most astrocytes act as a reserve force to amplify neuroinflammation by releasing cytokines, chemokines, and other inflammatory mediators. They upregulate multiple genes, augment cytoskeletal dimensions, extend their processes, increase glial fibrillary acidic protein expression, and enhance immunoreactivity, ultimately forming glial scars.^[19,20]^ In addition, activated astrocytes release Glu, and when excess Glu binds to the extrasynaptic NMDAR (N-methyl-d-aspartate receptor), brain-derived neurotrophic factor (BDNF) synthesis is reduced, affecting neuronal integrity.^[6,21]^ Stimulation of astrocytes surrounding the BBB by metabolic glutamate receptor-1 results in elevated Ca2^+^ levels. This, in turn, triggers arteriolar dilation, increases BBB permeability, and allows a more significant influx of peripheral inflammatory factors into the central region, thus intensifying the state of central nervous system inflammation.^[22]^

Furthermore, astrocytes, integral components of the neurovascular unit (NVU), provide neurotrophic support and promote the establishment and maintenance of synaptic activity and transmission. They regulate cerebral blood flow and influence certain functions and properties of the BBB and NVU.^[23]^ A1 astrocytes increase the levels of many genes in the classical complement cascade, such as C1r, C1s, C3, and C4, which are detrimental to NVU.^[24,25]^ As the pathological process advances, type A2 astrocytes become activated in conditions such as cerebral ischemia. These astrocytes upregulate the levels of neurotrophic factors, including transforming growth factor-β, among others. This upregulation serves to inhibit CNS inflammation, support neuronal survival, regulate myelination, and enhance the function of the extracellular matrix, significantly promoting the NVU remodeling.^[26]^ In conclusion, the beneficial and detrimental effects of astrocytes may be due to their heterogeneity and different phenotypes. The interplay between activated microglia and reactive astrocytes may lead to immune “optimization” through mutual communication and collaboration during neuroinflammation.^[18]^ We therefore believe that as technology advances, microglia-astrocyte interactions may become an effective and precise therapeutic target in the future.

### 2.3. Synapses and neurons

As previously indicated, astrocytes play a multifaceted role as closely interconnected cells with neurons and blood vessels. They communicate with neuronal pre- and postsynaptic terminals and facilitate the modulation of synaptic transmission by releasing neurotransmitters such as glutamate, D-serine and ATP.^[18,27]^ To illustrate, in vitro investigations involving primary hippocampal neurons have revealed that TNF signaling mechanisms regulate synaptic scaling by enhancing the trafficking of glutamate receptors and reducing the abundance of surface GABA receptors.^[28]^ Neuroimaging inquiries have consistently reported diminished volumes in the prefrontal cortex and hippocampus among patients diagnosed with major depression. These observations imply neuronal atrophy within these regions.^[29]^ Animal experiments demonstrate that animals modeling depression exhibit reduced dimensions of hippocampal neurons, processes, nuclear atrophy, and even neuronal loss, accompanied by a substantial reduction in synaptic density.^[30]^ Conversely, the administration of antidepressants has been shown to promote the formation of hippocampal neuron synapses and prevent neuronal atrophy, consequently mitigating depression-like behaviors.^[31]^ These findings collectively suggest that stress-induced microglial activation and the ensuing neuroinflammatory processes can initiate neurobiological consequences via cytokine receptor signaling. These consequences may influence neuroplasticity and contribute to the manifestation of depression-like behaviors.

## 3. Mechanism of polyphenols in preventing and treating depression through anti-neuroinflammation

Polyphenols are a class of phytochemicals found in natural plants, comprising over 8000 distinct structural variations, all characterized by multiple aromatic rings with hydroxyl groups.^[32]^ Based on their chemical composition, plant-synthesized polyphenols can be categorized into flavonoids and non-flavonoids.

Flavonoids represent the most abundant subgroup of polyphenols, with the majority sharing a common 1-diphenylpropane or 1-C6-C3-C6 skeleton structure.^[33]^ Broadly, flavonoids can be further categorized into 12 primary types, differentiated by their hydroxylation patterns and oxidation modes. Representative compounds within this category include flavonol, flavanone, anthocyanin, flavonoids, and isoflavones. These compounds are commonly found in various plant parts, including fruit seeds or pericarps (e.g., grapes, litchi, rambutans, mangosteen, and dragon fruits), vegetables (e.g., legumes, cereals, and cauliflower), and numerous herbs (e.g., Scutellaria baicalensis, ginkgo biloba, and wolfberry).^[34]^

On the other hand, non-flavonoids encompass phenolic acid, phenolic alcohol, stilbene, lignans, curcumin, and coumarin.^[33]^ Phenolic acids refer to compounds containing carboxylic acid groups attached to the benzene ring. They are primarily categorized into hydroxybenzoic acid (e.g., Gallic acid) and hydroxycinnamic acid (e.g., caffeic acid, ferulic acid).^[35]^ These compounds are commonly encountered in various edible plants as amides, esters, or glycosides, typically within plant seeds, pericarps, and vegetable leaves. Stilbenes are distinguished by their substitution of hydroxyl and methoxy groups across two benzene rings, although they are less prevalent than other polyphenols.^[32]^ Lignans, formed by the oxidative dimerization of multiple phenylpropane units, are typically found in aglycone, ester, or glycoside forms in various plant-based foods, including grains, vegetables, and fruits.^[36]^ Curcumin, diferulylmethane, is the principal active component derived from turmeric rhizomes.^[37]^ Imperatorin, alternatively recognized as [9-(3-methyl butyryl 2-acyloxy)-7H-furan [3jing2murg] chroene-7-one], is a naturally occurring coumarin compound widely distributed in medicinal plants like Angelica dahurica and Radix Glehniae.^[34]^

Table 1 provides specific examples of these natural polyphenols and describes some known food sources for each category.

**Table 1:** Food sources of major polyphenols.

Taxonomic		Polyphenol	Plant sources
Flavonoids	Flavonols	Quercetin	Black tea, green tea, red wine, white wine, walnut, almond, apple peel, blueberry, orange, dark chocolate, raw spinach, onion
	Flavones	Apigenin	Parsley, celery, oranges, grapes, citrus, perilla leaves
	Flavanones	Hesperidin	Citrus fruit
	Isoflavones	Genistein	Soybeans
	Anthocyanidins	Anthocyanins	Blueberry, cherry, black oak, purple grape and blackcurrant
Non-flavonoids	Phenolic acid	Chlorogenic acid	Wormwood, apples, cherries, tea
	Phenolic acid	Ferulic acid	Wheat, dark chocolate, dates
	Phenolic acid	Ellagic acid	Stem and bark of raspberries, pomegranates, aconite, eucalyptus and nuts
	Phenolic acid	Gallic acid	Grape seed, rose flowers, sumac, oak, and witch hazel
	Stilbenes	Resveratrol	Grape, black clover, mulberry and peanut
	Lignans	Schisandrin	Schisandra chinensis
	Curcumin	Curcumin	turmeric
	Coumadin	Imperatorin	Radix angelicae dahuricae dahurica

**Figure 2.**
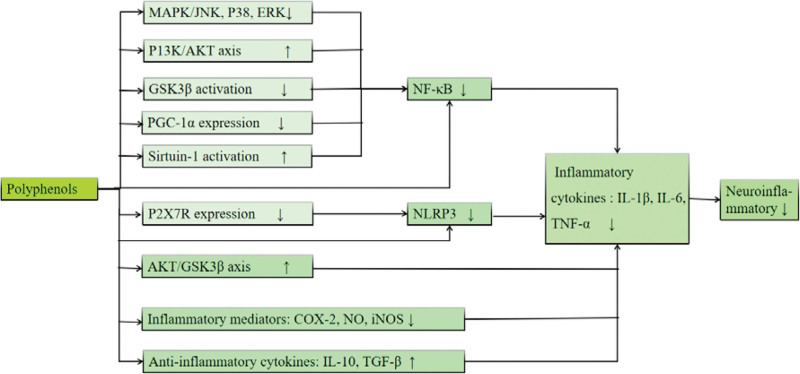
A schematic summary of potential anti-neuroinflammatory mechanisms of polyphenol extracts against depression.

Currently, polyphenols are integrated into human diets and nutritional and health products, displaying potential for a diverse array of beneficial mechanisms, encompassing antioxidation, free radical scavenging, anti-cancer properties, anti-inflammatory effects, cardiovascular protection, and neuroprotection, among others.^[38]^ Both clinical and preclinical experiments have demonstrated the significant role of polyphenols or specific polyphenol extracts, such as curcumin, apigenin, ellagic acid, chlorogenic acid, ferulic acid, quercetin, anthocyanin, resveratrol, and more, in exerting antidepressant effects.^[39–41]^ The absorption, utilization, and biological activities of ingested polyphenols are a subject of inquiry. Scientific evidence suggests that polyphenols face challenges in crossing the BBB and achieving therapeutic concentrations within the CNS. Nevertheless, they exert beneficial effects through extensive interactions with the gut microbiota (GM).^[42]^ Approximately 5% of dietary polyphenols are absorbed in the small intestine. In comparison, the remaining 90% to 95% continue to the colon, where they engage with the GM and communicate with the brain via the microbial community-gut-brain axis. This interaction helps to combat neuroinflammation, prevent neuronal apoptosis, and play a neuroprotective role.^[43]^ Subsequent sections will explore the specific mechanisms of several extensively studied polyphenols in their fight against neuroinflammation. This will provide a theoretical basis for the clinical use of polyphenols (refer to Table 2, Fig. 2).

**Table 2:** Specific mechanism of polyphenols against neuroinflammation.

Poly.phenol	Action pathway	Changes of inflammatory factors
Anthocyanidins	Inhibition of activation of NF-κB, P13/AKT and MAPKs pathways	iNOS↓;NO↓;PGE2↓; TNF-α↓;COX-2↓;1L-1β↓
Curcumin	Inhibit the activation of NF- kappa B; inhibit the activation of P2X7/NLRP3 inflammatory bodies; reduce the content of tryptophan in IDO and Kynurenine/; down-regulate the expression of p38MAPK and P13/AKT pathway	iNOS↓; COX-2↓
Quercetin	Reduce the expression of p38MAPK; regulate NF- kappa B/HO pathway; regulate HO-1/Nrf2 pathway	NO↓;iNOS↓
Ferulic acid	Inhibit the expression of NF- κ B, NLRP3 inflammatory bodies, and inhibit the phosphorylation of MAPK including P38 and JNK	TNF-α↓;1L-16↓;1L-1β↓
Resveratrol	Inhibit the activation of NF- κ B; up-regulate the expression of pCREB and BDNF; induce the activation of AKT/GSK3 β pathway; activate sirtuin-1 protein to inhibit the expression of NF- κ B and NLRP3	TNF-α↓;1L-16↓;1L-1β↓

### 3.1. Anthocyanins

Anthocyanins represent a significant subclass of flavonoids, primarily found in red fruits such as blueberries.^[44]^ Their high human intake, combined with their antioxidant and anti-inflammatory properties,^[44,45]^ position them as promising candidates for the prevention and treatment of conditions such as cardiometabolic disease, cancer, visual impairment and neurological disorders.^[46]^ Moreover, anthocyanins have been shown to yield favorable effects in addressing neuroinflammation.

The results of the study found that anthocyanin-rich fruit pulp fractions protected microglia exposed to lipopolysaccharide (LPS), while significantly inhibiting p38 and NF-κB activation, and decreasing levels of iNOS (inducible nitric oxide synthase), COX-2 (Cyclooxygenase-2) and TNF-α.^[47]^ In a separate study, anthocyanins extracted from black bean seed coats were observed to thwart the LPS-induced activation of NF-κB, PI3K/Akt and MAPK signaling cascades in microglia. This led to a reduction in the production of proinflammatory mediators, including nitric oxide (NO), prostaglandin E2, TNF-α, and interleukin IL-1β.^[48]^ Additionally, the anthocyanin extract from C. persica exhibited protective effects against memory deficits and neuroprotection in LPS-exposed mice. It achieved this by restoring IL-1β, TNF-α, and IL-1 levels in the hippocampus, reducing microglial and astrocyte activation in the cortex and hippocampus, and inhibiting peripheral immune cell infiltration in the same brain regions.^[49]^ Similarly, Khan et al reported that anthocyanins from soybean seed coats reduced inflammatory markers NF-κB, TNF-α, and IL-1β in the hippocampus and ameliorated memory impairment in LPS-treated mice.^[50]^ Furthermore, evidence suggests that anthocyanins may modulate neuronal cell death signaling pathways, regulate mitochondrial function, inhibit protein aggregation, enhance autophagy, and prevent excitotoxicity-induced neuronal cell death by maintaining calcium homeostasis.^[51]^ In conclusion, anthocyanins can reduce the production of pro-inflammatory mediators by preventing the activation of the NF-κB, PI3K/Akt and MAPKs signaling cascades in microglia; they can also restore the levels of anti-inflammatory factors in the hippocampus to reduce the activation of glial cells and astrocytes to prevent the infiltration of immune cells, thus playing a neuroprotective role.

### 3.2. Curcumin

Curcumin is a hydrophobic polyphenol belonging to the flavonoid group, derived mainly from the rhizomes of the curcuma plant, which is used in the production of the spice curcuma. It acts on numerous transcription factors, pro-inflammatory cytokines, growth factors, protein kinases and various enzymes, resulting in a spectrum of biological effects, particularly anti-inflammatory properties.^[51]^ In the context of significant depression, curcumin orchestrates multiple neurobiological mechanisms intertwined with the pathophysiology of depression. These mechanisms encompass aspects associated with monoaminergic neurotransmission, hypothalamic-pituitary-adrenal axis functionality, excitotoxicity, neuroplasticity, and inflammatory pathways.^[52,53]^

At the molecular level, curcumin is a natural inhibitor of NF-κB, thereby regulating the expression of many genes, including those under the control of NF-κB. These genes include COX-2, matrix metalloproteinase-9, iNOS, interleukin-8 and anti-apoptotic proteins.^[54]^ In a study involving rats subjected to chronic mild and unpredictable stress (CUMS), the animals exhibited depression-like behavior, heightened IDO activity, increased mRNA expression of inflammatory factors (IL-1β, IL-6, TNF-α), activation of the NF-κB signaling pathway, and stimulation of the P2X7R/NLRP3 inflammasome axis, coupled with reduced concentrations of 5-HT in the hippocampus. Curcumin effectively reversed these behavioral and neuroimmune aberrations induced by CUMS, leading to a notable decrease in mRNA expression of pro-inflammatory cytokines and the inhibition of NF-κB activation.^[55]^ Furthermore, curcumin curtailed the stress-induced activation of the P2X7R/NLRP3 inflammasome axis, consequently diminishing the conversion of IL-1β to its mature form. The addition of curcumin also attenuated the stress-induced activation of IDO and the increase in the kynurenine/tryptophan ratio. These collective changes underpin, at least in part, the antidepressant effects of curcumin, suggesting a link between these effects and the inhibition of NLRP3 inflammasomes and related pathways. In addition, curcumin demonstrated the ability to reduce depression-like behavior and neuronal apoptosis by downregulating the IL-1β/p38 MAPK pathway in the ventromedial prefrontal cortex of rats.^[56]^ Further studies by Wang et al^[57]^ demonstrated that curcumin has the ability to inhibit lipopolysaccharide-induced microglial activation, the NF-κB signaling pathway, and the up-regulation of pro-inflammatory cytokines. It also reduced the lipopolysaccharide-induced elevation of iNOS and COX-2 mRNA levels in the mouse hippocampus and prefrontal cortex. Curcumin at concentrations of 10, 30, 40, and 50 μM attenuated inflammatory responses in lipopolysaccharide-stimulated microglia within the PI3K/Akt pathway. These results suggest that curcumin plays a central role in mitigating glial cell suppression by down-regulating the PI3K/Akt network.^[58]^

### 3.3. Quercetin

Quercetin is a flavonol phytochemical, recognized as the most abundant flavonoid present in a variety of vegetables and fruits, including onions, broccoli, berries, grapefruits, apples, black and green tea, red grapes, citrus fruits, green leafy vegetables, and beans.^[59,60]^ It is widely acknowledged that quercetin has many health benefits, including reducing the risk of neurological diseases, cancer, cardiovascular conditions, allergies, and more. Additionally, it has both anti-inflammatory and antioxidant properties.^[61]^ Its immunomodulatory properties include the potential to downregulate cytokine secretion, modulate the expression of inflammatory genes and influence signaling pathways, all of which have been shown to have neuroprotective effects.^[62,63]^

Mehta et al^[64]^ has elucidated the underlying neurobiological mechanisms responsible for the antidepressant effects of quercetin within the context of chronic, unpredictable stress models of depression. Mice exposed to chronic unpredictable stress (CUS) exhibit anxiety, depression-like behavior and cognitive impairment. CUS also increases the presence of inflammatory molecules and markers of oxidative stress (e.g. thiobarbituric acid-reactive substances, nitric oxide), while decreasing levels of antioxidants (total thiols, catalase) in the mouse hippocampus, leading to hippocampal neuronal damage. Nevertheless, quercetin therapy alleviates CUS-induced behavioral, cognitive and neurological impairments in mice by inhibiting neuroinflammatory mediators and oxidative stress in the brain. Other studies have also shown that quercetin alleviates depression-like behavior in rodents exposed to chronic, unpredictable mild stress by downregulating pro-inflammatory cytokines (IL-1β, TNF-α)^[65]^ and inflammatory mediators (nitric oxide, inducible nitric oxide synthase).^[66]^ The study investigated the antioxidant properties of quercetin and reported a strong link between the protective effects of quercetin and the MAPK pathway, finding that quercetin increased the expression of p38MAPK in LPS-stimulated microglial cells, inducing oxidative stress, which in turn inhibited NO release.^[67]^ Additionally, quercetin suppresses NO and iNOS expression by modulating the NF-κB/HO pathway in lipopolysaccharide-exposed microglia.^[68,69]^ In a zebrafish model, quercetin dampens inflammatory responses triggered by 6-hydroxydopamine toxicity by inhibiting the TNF-α pathway.^[70]^ Furthermore, quercetin reduces lipopolysaccharide-induced TNF and IL-1 mRNA expression in glial cells and astrocytes and mitigates microglial and microglia-mediated neuronal cell death.^[71,72]^ It also eases activated astrocytes and prevents neuroinflammation induced by zidovudine in the central nervous system.^[73]^ Activated astrocytes and microglia mediate the activation of cytokines and reactive oxygen species, which in turn affect neurons and trigger neuronal degeneration.^[74]^ Quercetin has been reported to attenuate manganese-induced neurotoxicity by preventing neuroinflammation-mediated neurodegeneration through the regulation of heme oxygenase-1/nuclear factor erythroid 2-related factor 2 and NF-κB pathways.^[75]^ In addition, quercetin has demonstrated neuroprotective potential against neurodegeneration in a variety of in vitro and in vivo mouse models.^[72,76]^

In conclusion, quercetin undeniably confers benefits on the human body through the above mechanisms. However, it’s worth noting that quercetin can have opposite effects at different concentrations. Lower concentrations of quercetin have been shown to improve fish behavior and fitness, whereas higher concentrations (up to 1000 μg/L) have been shown to have negative effects on these behaviors.^[77]^ Consequently, future research should focus on determining the optimal quercetin concentration for mitigating inflammation, promoting synaptic and neuronal growth, and exploring the combined use of quercetin with other drugs or polyphenols.

### 3.4. Ferulic acid

Ferulic acid, a derivative of cinnamic acid, is present in various plants, including vegetables, fruits, wheat, cereals, and barley. It is also a component of traditional Chinese medicines such as Angelica sinensis, Chuanxiong, Cimicifuga racemosa, Coptis chinensis, and Zizyphus jujuba.^[77]^ Ferulic acid exhibits numerous advantageous biological activities, encompassing antioxidant, anti-allergic, hepatoprotective, anticancer, antibacterial, and anti-inflammatory properties.^[78]^ It impacts the expression of various pro-inflammatory cytokines, pro-apoptotic signaling pathways, and diverse neural signaling mechanisms through interactions with multiple receptors or enzymes.^[79]^

In a CUMS depression model, ferulic acid treatment has been observed to suppress microglial activation, NF-κB signaling, and NLRP3 inflammation. This intervention reverses the activation of pro-inflammatory cytokines and reduces the mRNA expression of IL-1β, IL-6, and TNF-α in the prefrontal cortex of mice subjected to CUMS-induced depression.^[80]^ These findings suggest that the antidepressant effects of ferulic acid are mediated through anti-inflammatory mechanisms in the CUMS-induced mouse model. Moreover, ferulic acid has been shown to inhibit IL-6 expression by reducing the levels of phosphorylated IKK (inhibitor of kappa B kinase) and hindering the nuclear translocation of NF-κB. This, in turn, reduces the transcriptional activity of IL-6 driven by the NF-κB-binding site.^[81]^ Furthermore, ferulic acid pretreatment has been found to inhibit the phosphorylation of MAPKs, including p38 and JNK, in bovine endometrial epithelial cells during the inflammatory response induced by LPS. This inhibition reduces pro-inflammatory cytokine production, including IL-1β, IL-6, TNF-α, and IL-8.^[82]^ Ferulic acid and its derivatives can cross the BBB at sufficient concentrations, giving rise to neurotherapeutic effects.^[79,82]^ In a study by Xu,^[83]^ ferulic acid was evaluated for its antidepressant-like activity using a reserpine-induced mouse model. Ferulic acid was found to facilitate behavioral disturbances by up-regulating monoamine neurotransmitters in mouse brains’ hippocampus and frontal cortex. Additionally, higher doses of ferulic acid treatment led to increased levels of 5-HT and norepinephrine in the hypothalamus and down-regulation of IL-1β, TNF-α, nitrite, and lipid peroxidation. Furthermore, ferulic acid was observed to elevate the levels of BDNF, PSD95, and synaptic protein I in the hippocampus of mice, promoting hippocampal neurogenesis and thereby enhancing cognitive function.^[84]^

Recent pharmacological studies on ferulic acid have revealed its broad applications in combating oxidative stress, inflammation, vascular endothelial injury, fibrosis, apoptosis and more. Its potential to improve bowel motility and reduce medication-related discomfort, combined with its synergistic effects with conventional antidepressants, make it a promising new “triple reuptake inhibitor” for future development.

### 3.5. Resveratrol

Resveratrol naturally occurs in various nutrient-rich foods, including grapes, peanuts, blueberries, bilberries, cranberries, purple grape juice, and polygonum cuspidatum.^[84]^ It has demonstrated the ability to down regulate pro-inflammatory mediators, transcription factors, and signaling pathways that influence the expression of inflammatory responses.

In a prior investigation by Ge,^[85]^ the molecular mechanism underlying the antidepressant effect of resveratrol in LPS-induced depression was explored. This effect was partially attributed to the downregulation of NF-κB signaling, the reduction of pro-inflammatory cytokine levels, and the upregulation of phosphorylated cAMP response element-binding protein and BDNF expression in mouse brains. The etiology of depression also involves neuroinflammation and apoptosis induced by the AKT/GSK3β signaling pathway. Shen’s research^[86]^ proposed that the antidepressant effect of resveratrol in CUMS-treated rats is mediated by the induction of the AKT/GSK3β signaling pathway and the subsequent downregulation of pro-inflammatory cytokines. Liu^[87]^ examined the anti-neuroinflammatory effects of resveratrol in reducing anxiety and depression-like behavioral symptoms induced by ovariectomy (OVX). In this study, male mice subjected to OVX surgery exhibited behavioral disturbances associated with depression, increased microglial activation, mRNA expression of NLRP3 and NF-κB, and decreased mRNA expression of sirtuin-1 in the hippocampus. Administration of resveratrol to these mice mitigated behavioral and neuroinflammatory abnormalities induced by OVX surgery. Sirtuin-1 is a member of the sirtuin protein family known for deacetylating histones and critical transcription factors, resulting in the transcriptional repression of numerous inflammation-related genes. It plays a pivotal role in regulating inflammation. A previous study showed that resveratrol activated sirtuin-1 in OVX mice, reducing anxiety and depression-like behaviors by inhibiting the hippocampus’s NF-κB and NLRP3 signaling pathways, thereby preventing neuroinflammatory processes.^[88]^

Resveratrol’s limited water solubility and poor body absorption lead to low oral bioavailability. Consequently, the drug can be encapsulated in nanocarriers to enable sustained release and targeting. This approach can help reduce the potential toxic side effects of the drug.^[89]^ As contemporary pharmaceutics advances and related disciplines develop, preparation technology continuously innovates and optimizes. An intelligent drug delivery system that integrates protection, targeting, and controlled release can be created by considering resveratrol’s physical and chemical properties and clinical requirements. Such a system can better leverage the antidepressant efficacy of resveratrol in clinical practice.

## 4. Conclusion

Given the increasing incidence of depression, there is a growing need for a more targeted approach to mitigate risk factors in developing the condition and managing depressive symptoms. Neuroinflammation emerges as a potential therapeutic target that affects a broad spectrum of neural structures and functions, contributing to the pathophysiology and progression of depression. This article provides an overview of findings from preclinical animal studies that investigate the role of polyphenols in addressing neuroinflammatory pathways associated with depression. The research suggests that polyphenols can modulate the expression of pro-inflammatory cytokines through direct interference with inflammatory regulators such as NF-kB and NLRP3 inflammasomes. Alternatively, they can downregulate the expression of pro-inflammatory cytokines by influencing protein kinases or modulating the activity of signaling pathways triggered by protein kinases (including MAPK pathways such as JNK, p38, and ERK1/2, AKT/GSK3β pathways, P2X7R as well as PI3K/Akt pathways). These pro-inflammatory cytokines include IL-1, IL-6, IL-18, and TNF-α. In addition, polyphenols can influence the reduction of inflammatory mediators (NO, iNOS, COX-2) and the synthesis of anti-inflammatory cytokines (1L-10, TGF-β), which can attenuate neuroinflammation, reduce neuronal apoptosis and exert neuroprotective effects. Given the promising anti-neuroinflammatory potential of polyphenols, they may have significant potential in the treatment of treatment-resistant depression.

Pharmaceuticals or plant-derived polyphenols offer the prospect of identifying novel anti-inflammatory candidates. Potential lead compounds can be selected from anti-inflammatory agents and subjected to structural modification or engineering to enhance their anti-inflammatory efficacy and stability, offering more excellent prospects for drug development. However, several questions remain to be addressed. Firstly, polyphenols can be combined with probiotics or undergo structural modifications to enhance their bioavailability and absorption rate, improving their utility as nutritional supplements. Secondly, clinical trials pose some challenges, including small sample sizes, brief trial durations, the lack of a consensus on the safe and effective dosages for human use, and the potential for multiple confounding factors to influence the determination of clinical efficacy during treatment. Thirdly, a systematic and comprehensive evaluation of polyphenols is essential during development, encompassing effectiveness, safety, cost-effectiveness, patient compliance, and label information considerations. Fourthly, the combined application of various plant polyphenols and the concurrent use of polyphenols with antidepressant medications may yield more effective treatments for depression. Lastly, further exploration of the mechanisms of action of polyphenols is needed to develop a comprehensive network system, providing a more robust scientific foundation for the prevention and treatment of depression.

## Acknowledgments

The author sincerely thanks every teacher and student in Professor Yan Yang’s team of Chengdu University of Traditional Chinese Medicine for their help.

## Author contributions

**Conceptualization:** Yuting Guo, Yan Yang.

**Funding acquisition:** Yan Yang.

**Writing – original draft:** Yuting Guo.

**Writing – review & editing:** Yan Yang.
